# Synergistic effects of type I PRMT and PARP inhibitors against non-small cell lung cancer cells

**DOI:** 10.1186/s13148-021-01037-1

**Published:** 2021-03-10

**Authors:** Claudia Dominici, Nicolas Sgarioto, Zhenbao Yu, Laura Sesma-Sanz, Jean-Yves Masson, Stéphane Richard, Noël J.-M. Raynal

**Affiliations:** 1 Segal Cancer Center, Lady Davis Institute for Medical Research and Gerald Bronfman Department of Oncology and Departments of Biochemistry, Human Genetics and Medicine, McGill University, Montréal, QC H3T 1E2 Canada; 2 Département de Pharmacologie et Physiologie, Université de Montréal, and Research Centre of the Sainte-Justine University Hospital, Montréal, QC H3T 1C5 Canada; 3 Genome Stability Laboratory, CHU de Québec Research Center, Oncology Division, Department of Molecular Biology, Medical Biochemistry and Pathology, Laval University Cancer Research Center, 9 McMahon, Québec, QC G1R 3S3 Canada

**Keywords:** Type I PRMT inhibitors, PARP inhibitors, MTAP, Drug resistance, Synergy, Non-small cell lung cancer cells, DNA damage, And cytotoxic

## Abstract

**Background:**

Non-small cell lung carcinoma (NSCLC) is a leading cause of cancer-related death and represents a major health burden worldwide. Current therapies for NSCLC include chemotherapy, immunotherapy, and targeted molecular agents such as tyrosine kinase inhibitors and epigenetic drugs such as DNA methyltransferase inhibitors. However, survival rates remain low for patients with NSCLC, especially those with metastatic disease. A major cause for therapeutic failure is drug resistance, highlighting the need for novel therapies and combination strategies. Given that epigenetic modulators such as protein arginine methyltransferases (PRMTs) are frequently overexpressed in cancers, PRMT inhibitors are a promising class of cancer therapeutics. We screened a library of epigenetic and anticancer drugs to identify compounds that would synergize with MS023, a type I PRMT inhibitor, in decreasing the viability of methylthioadenosine phosphorylase (MTAP)-negative NSCLC cells.

**Results:**

Among 181 compounds, we identified PARP inhibitors (PARPi) as having a strong synergistic interaction with type I PRMT inhibition. The combination of MS023 and the PARP inhibitor BMN-673 (Talazoparib) demonstrated strong synergistic interaction at low nanomolar concentrations in MTAP-negative NSCLC cell lines A549, SK-LU-1 and HCC4006. The re-introduction of MTAP decreased the sensitivity of the combination therapy in A549. The combination therapy resulted in elevated γ-H2AX foci indicating increased DNA damage causing decreased cell viability. Lastly, the combination therapy was effective in PARPi resistant ovarian cancer cells, suggesting that type I PRMT inhibitors could mitigate PARPi resistance, thus potentially having an important clinical impact for cancer treatment.

**Conclusions:**

These findings identify a novel cancer drug combination therapy, which is more potent than the separate single-agent therapies. Thus, combining PARP inhibitors and type I PRMT inhibitors represents a new therapeutic opportunity for MTAP-negative NSCLC and certain cancer cells resistant to PARP inhibitors.

**Supplementary Information:**

The online version contains supplementary material available at 10.1186/s13148-021-01037-1.

## Background

Lung cancer is the leading cause of cancer-related death worldwide and is highly taxing on health care systems [1]. The vast majority (about 85%) of lung cancer cases are of the non-small cell lung cancer (NSCLC) subtype [2]. Early-stage NSCLC is addressed with surgery, however later stages NSCLC present a challenge that is not adequately met with current therapeutics [3, 4]. Myriad oncogenic pathways have been identified in patients with NSCLC with varying degrees of penetration, such as aberrations in receptor tyrosine kinase signalling, mTOR signalling, and components of the cell cycle [4, 5]. While deficiencies in these well-studied pathways create an opening for targeted molecular therapies, the complex etiology of NSCLC presents a challenge in developing a unified therapy that can be extended to a broad range of patients. Targeting the epigenetic regulation with small molecule inhibitors provides a promising avenue in the development of successful therapies. Indeed, inhibitors of epigenetic modulators are actively being pursued [6, 7]. Unfortunately, single-agent delivery of epigenetic inhibitors has been met with limited success due to high toxicity or impermanent effects [8, 9]. Therefore, delivering combinations of synergic epigenetic inhibitors at lower doses represents a suitable alternative. In fact, the success observed with targeting DNA methyltransferases (DNMTs) in combination with histone deacetylases (HDACs) in NSCLC provides a rationale for uncovering other combinations of epigenetic modifiers, which can be developed into effective therapies [10, 11].

Arginine methylation is an abundant post-translational modification identified in many proteins including histones, RNA-binding proteins, transcription factors and their coregulators, and DNA damage repair proteins [12]. Arginine methylation is mediated by a family of nine protein arginine methyltransferases (PRMTs) [13]. PRMTs transfer methyl groups from S-adenosylmethionine (SAM) to the guanidino nitrogens of arginine, generating methylated arginines and S-adenosylhomocysteine (SAH) as a by-product. PRMTs are divided into three subtypes [13]: type I catalyzes the formation of monomethylarginine (MMA) before a dimethylation reaction to produce asymmetric dimethylarginine (aDMA). PRMT1 is the type I enzyme responsible for the majority of aDMA. Type II enzymes also generate MMA as an intermediate for the production of symmetric dimethylarginine (sDMA), with PRMT5 being the major enzyme generating this modification. PRMT7 is only known type III enzyme only able to generate MMA [14]. The early discovery of a transcriptional co-activator function of PRMT1 and PRMT4 (CARM1) linked arginine methylation to the field of epigenetics [15, 16]. In addition, arginine methylation is known to be linked to double-strand DNA break (DSB) repair through methylating the DNA damage proteins and affecting cell cycle checkpoints [17–20].

Overexpression of PRMTs resulting in altered methylarginine patterns is a common feature of cancer cells [21], and therefore PRMTs may serve as key therapeutic targets for intervention [12]. Promising small-molecule inhibitors exist for many PRMTs [22–26] and are in clinical trials. Notably, the type I PRMT inhibitor GSK3368715 is in phase I clinical trials for the treatment of diffuse large B-cell lymphoma and solid tumors (clinicaltrials.gov identifier number: NCT03666988). Additionally, PRMT5 inhibitors JNJ-64619178 and GSK3326595 are currently in phase I clinical trials (ClinicalTrials.gov identifier numbers NCT03573310 and NCT03614728, respectively) for patients with advanced cancers.

Substrate scavenging exists between type I and II PRMTs, leading to a global increase in MMA and sDMA, when PRMT1 is deleted [27]. This interplay between type I and type II PRMTs has recently been shown to have therapeutic value for cancer treatment and thus, it is not surprising that PRMT1 is synthetic lethal to PRMT5 deletion [24, 28, 29]. Since PRMTs require SAM as a methyl donor, they are inextricably linked to methionine metabolism. The enzyme 5-methylthioadenosine phosphorylase (MTAP) is a key component of the methionine salvage pathway. Due to its genomic proximity to the tumor suppressor genes *CDKN2A* and *CDKN2B,* the *MTAP* gene is commonly co-deleted in human cancer. This genetic deletion is found in ~ 40% of NSCLC patients [30]. Interestingly, *MTAP*-deleted cancer cells accumulate the metabolite methylthioadenosine (MTA), which is a high-affinity inhibitor of PRMT5 activity [31–33]. *MTAP*-negative A549 cells have elevated levels of MTA, resulting in endogenous PRMT5 inhibition [31–33]. As a result, inhibition of PRMT1 with the recently developed inhibitor of type I PRMTs, MS023 [23] or GSK3368715, is inherently optimal at reducing cell viability of *MTAP*-negative cells such as A549 [24, 28, 29].

Herein, we aimed to identify compounds that synergized with MS023 and elevated its cytotoxicity in the A549 NSCLC cell line. A library of epigenetic and anticancer compounds was screened, and we measured viability in the presence or absence of MS023. We identified several poly(ADP)–ribose polymerase (PARP) inhibitors that had a strong synergistic interaction with type I PRMT inhibition in A549 cells. This synergistic effect was partially attenuated in A549 cells stably transfected with MTAP expressing lentiviral vectors. Furthermore, we show that MS023 and the PARP inhibitor BMN-673 (Talazoparib) also synergized in SK-LU-1 and HCC4006 NSCLC cell lines. The combination therapy produced a significant increase in the accumulation of γ-H2AX foci indicating increased DNA damage, which was responsible for decreased viability. Furthermore, we show that MS023 lessened the resistance to PARPi in the PEO ovarian cancer cell line, indicating a degree of robustness to the synergy of BMN-673 and MS023 in cancer cells. Our data identify a new combination therapy using type I PRMT (MS023) and PARP (BMN-673; Talazoparib) inhibitors to effectively promote cell death of NSCLC.

## Results

### Cell viability screen with epigenetic/anticancer library identifies compounds that synergize with the type I PRMT inhibitor, MS023

**Fig. 1:**
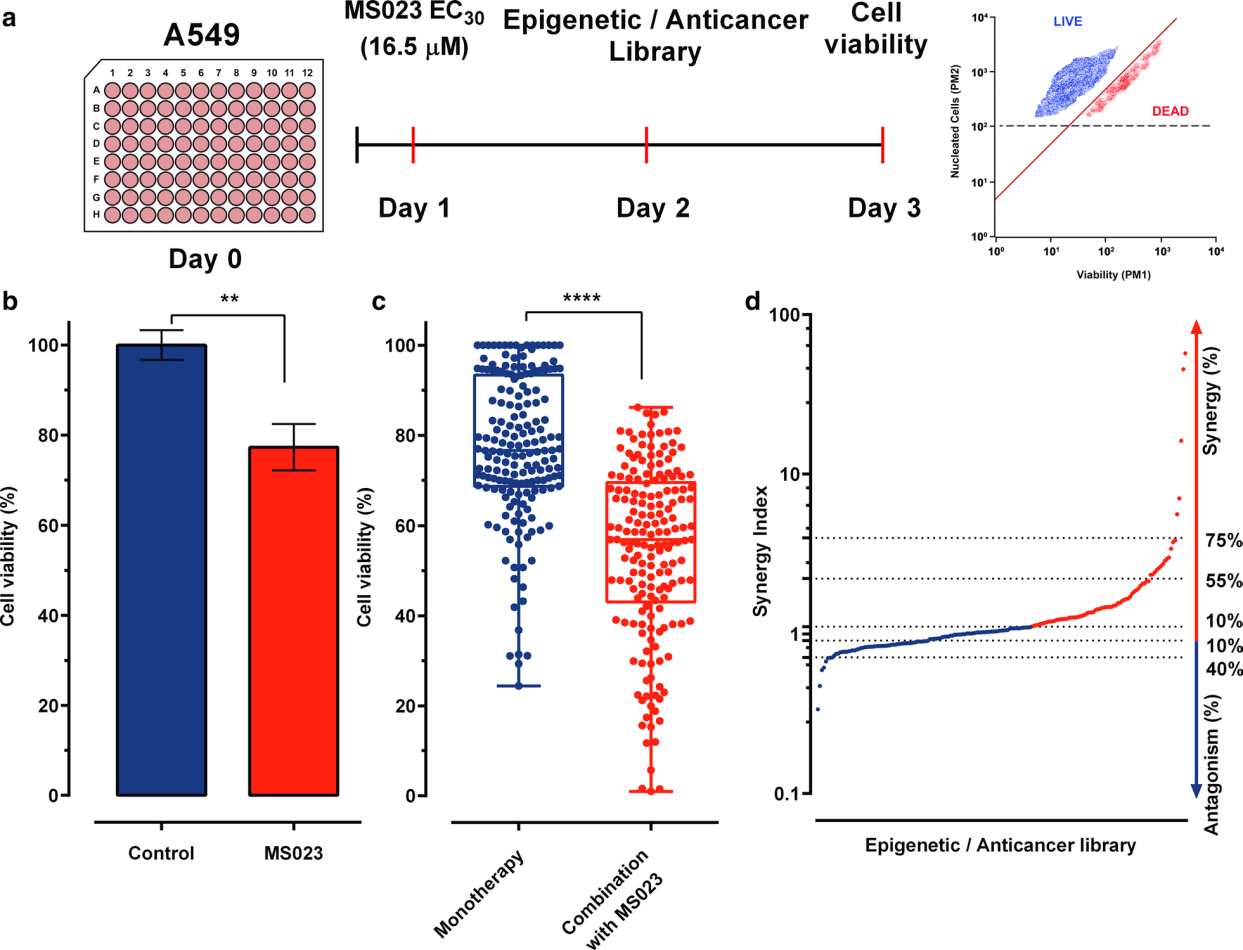
Cell viability screen to identify compounds targeting epigenetic regulators that synergize with MS023. **a** Scheme of the method used to measure synergy of MS023 with drugs from the Epigenetic/Anticancer compound library. Plates were seeded (20 K cells/well) and treated the next day with MS023 or DMSO. Drugs from the Epigenetic/Anticancer compound library were added on the following day. On day 3, viability was analyzed by flow cytometry using Guava® ViaCount™ Reagent. **b** Cell viability of A549 cells treated with DMSO (blue) or MS023 16.5 µM (red) through the screen. Cell viability was expressed as a percentage relative to DMSO-treated cells. Treatment with MS023 significantly (*p* = 0.0034 Wilcoxon matched-pairs signed rank test) decreased cell viability by 23% between vehicle condition (DMSO: 100.0 ± 3.3; *n* = 12) and MS023 treatment (77.3 ± 5.2; *n* = 11). **c** Drug screening results showing the distribution of viability of A549 cells after treatment with the drug library in monotherapy (10 µM, 24 h) or with a 24 h pre-treatment with MS023 (16.5 µM). Each dot represents cell viability (%) of a compound for each condition (monotherapy vs combination with MS023) relative to vehicle treated cells. Mean viability is significantly decreased by 23% (77.2 ± 16.4 vs. 54.2 ± 19.4; unpaired, nonparametric, Mann–Withney analysis, *p* < 0.001) indicating a global effect of MS023 pre-treatment on cell viability. For this representation, 3 drugs (CX-6258-HCL; Pirarubicin; MC1568) produced 1% cell viability and were excluded for the analyses in both conditions. **d** Synergy index obtained after the sequential combination of the MS023 followed by the Epigenetic/Anticancer drug library. A synergy index of 1.12 was set as a threshold for all combination to be considered synergistic

Since PRMT overexpression has been implicated in various human cancers and deficiency of PRMT activity can inhibit cancer cell proliferation and lead to cell death [21], we initiated a small molecule library screen to identify epigenetic/anticancer drugs, which can cause synthetic lethality in combination with MS023, an inhibitor of type I PRMTs [23]. The ChemSelleck Epigenetic compound library is composed of 181 compounds with epigenetic and anticancer activities that is divided into 6 main families; angiogenesis (2.2%), cell cycle (3.9%), cell signalling (10.5%), JAK/STAT (12.7%), DNA damage (17.1%), and epigenetics (53.6%). The cell line chosen for screening was A549, a human *MTAP*-negative NSCLC cell line (Fig. 1a).

We first performed multiple dose–response experiments after a 24 h MS023 exposure and measured dose-dependent cell viability inhibition. We selected 16.5 μM as the concentration of MS023 to be used for the screen, which resulted in a 23% decrease in A549 cell viability (Fig. 1b). For drug screening, A549 cells were pre-treated with 16.5 μM MS023 for 24 h, and then exposed for an additional 24 h to the drug library at 10 µM. Cell viability was measured by flow cytometry using Viacount reagent, which distinguishes between viable, pre-apoptotic and dead cells based on the differential permeability of DNA-binding dyes. For all screened compounds, the mean cell viability was 77.2 ± 16.4% in monotherapy (Fig. 1c). Pre-treatment with MS023 (16.5 µM, 24 h) followed by epigenetic/ anticancer compounds (10 µM, 24 h) produced a global and significant 25% reduction (*p* < 0.001) in cell viability to 54.2 ± 19.4%, indicating a general sensitization effect of MS023 (Fig. 1c). We then calculated a synergy index displaying quantitatively synergistic and antagonistic drug interactions with MS023 by normalizing cell viability of drug combination data to cell viability of each compound alone, allowing result comparison between each drug combination (Fig. 1d). We observed that 58 (42%), 13 (10%), and 5 (3%) compounds produced synergistic synthetic cell death by more than 10%, 55%, and 75%, respectively. Antagonist interactions by more than 10 and 40%, were produced by 47 (30%) and 8 (4%) compounds, respectively (Fig. 1d). All drug screening data are shown in Additional file 1: Table 1. Confirmation experiments of 40 compounds (20 most active and 20 less active compounds) showed a validation rate of 70% (28/40; Additional file 1: Figure S1 and Table 2). These experiments indicate that *MTAP*-deficient A549 NSCLC cells are sensitized to epigenetic/anticancer drugs following type I PRMT inhibitor pre-treatment.

**Fig. 2:**
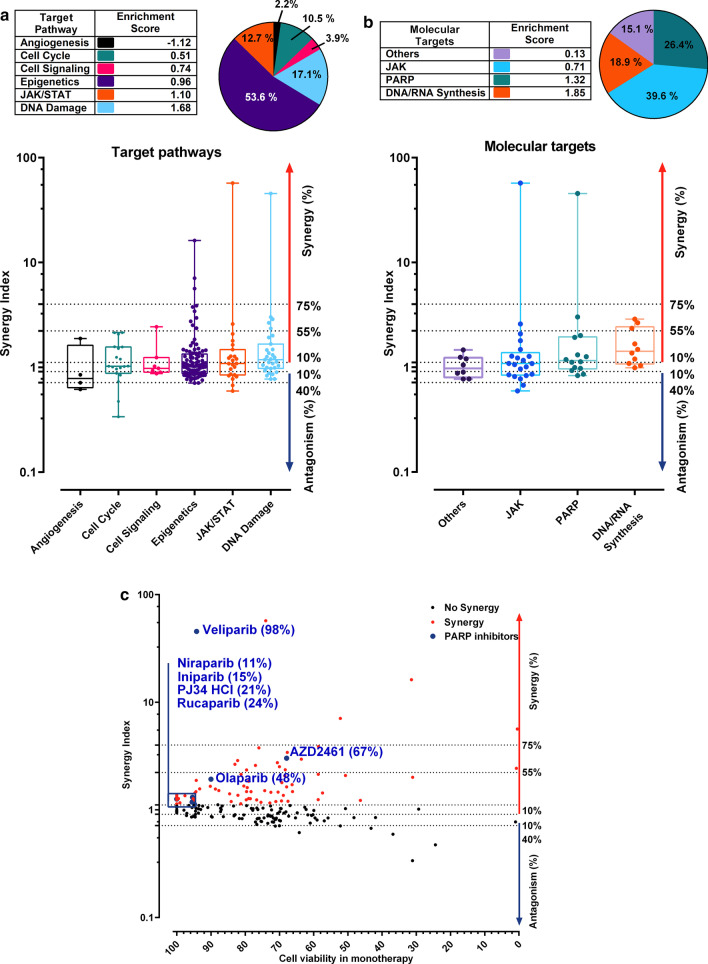
Drug screening analyses by target and molecular pathway reveal the synergistic interaction between MS023 and PARP inhibitors. **a** Synergy Index distribution of the drugs classified by target pathways. Table indicates the enrichment score of each family after the combination screen. Pie chart shows the repartition of each family in the library. Violin plots are classified by target pathways and ordered by enrichment score. Compounds associated with “JAK/STAT” and “DNA Damage” pathways respectively present an enrichment score of 1.10 and 1.68, respectively, suggesting a synergistic interaction with MS023. **b** Synergy Index distribution of the drugs classified by Molecular targets. Table indicates the enrichment score of each target family within the screen. Pie chart shows the repartition of each family in the library. Violin plots are classified by Molecular targets and ordered by enrichment score. Compounds inhibiting “PARP” and “DNA/RNA Synthesis” present an enrichment of 1.32 and 1.85 respectively, and seems more prone to synergise with MS023. **c** Synergy indexes of each compound are plotted in function of their cell viability in monotherapy. PARP inhibitors are indicated in the graph

To identify pharmacological effects from groups of drugs with similar targets, we evaluated the synergy index by regrouping drugs based on target pathways (Fig. 2a), or molecular targets (Fig. 2b). Pie charts are showing the percentage of drugs among each target pathways and molecular targets (Fig. 2a, b). Within each target pathway or molecular target sub-groups, we calculated an enrichment score per class by normalizing the effect of each sub-group to the number of compounds per category. Thus, the enrichment score takes into account the percentage of synergy of each compound and its own weight in its sub-group. An enrichment score above 1 suggests a category that synergizes with MS023. When classified by target pathways, mean synergy index per class showed that compounds belonging to DNA damage pathway (enrichment score = 1.68) were the most synergistic with MS023 followed by JAK/STAT (enrichment score = 1.10). In contrast, epigenetics (enrichment score = 0.96), cell signaling (enrichment score = 0.74), cell cycle (enrichment score = 0.51), and angiogenesis (enrichment score = −1.12) drug classes had low enrichment scores (< 1) and low to antagonistic synergistic index (Fig. 2a).

The two most enriched target pathways were DNA damage and JAK/STAT. When grouping based on molecular targets, compounds targeting DNA/RNA synthesis had the highest mean synergy index (enrichment score = 1.85) followed by PARP inhibitors (PARPi, enrichment score = 1.32; Fig. 2b). Interestingly, approved PARPi (Veliparib, Niraparib, and Olaparib) and PARPi in development (Iniparib, PJ34 HCl, and AZD2461) showed synergistic interactions with MS023, without causing cytotoxicity in monotherapy, suggesting a potentiation effect (Compounds that potentiate the effect of MS023 are found on the left-hand side of the graph, Fig. 2c). Overall, our screen in the *MTAP*-negative A549 NSCLC cell line showed that PARPi exhibited a high synergistic interaction with a type I PRMT inhibitor, which may be easily combined in a clinical setting to increase therapeutic efficacy (Fig. 2a–c). Observing enrichment in this drug class prompted further investigation into the synergistic potential between PARPi and the type I PRMT inhibitor MS023.

### Combination of MS023 with low concentrations of the PARP inhibitor BMN-673 (Talazoparib) results in synthetic lethality of A549 NSCLC cells

**Fig. 3:**
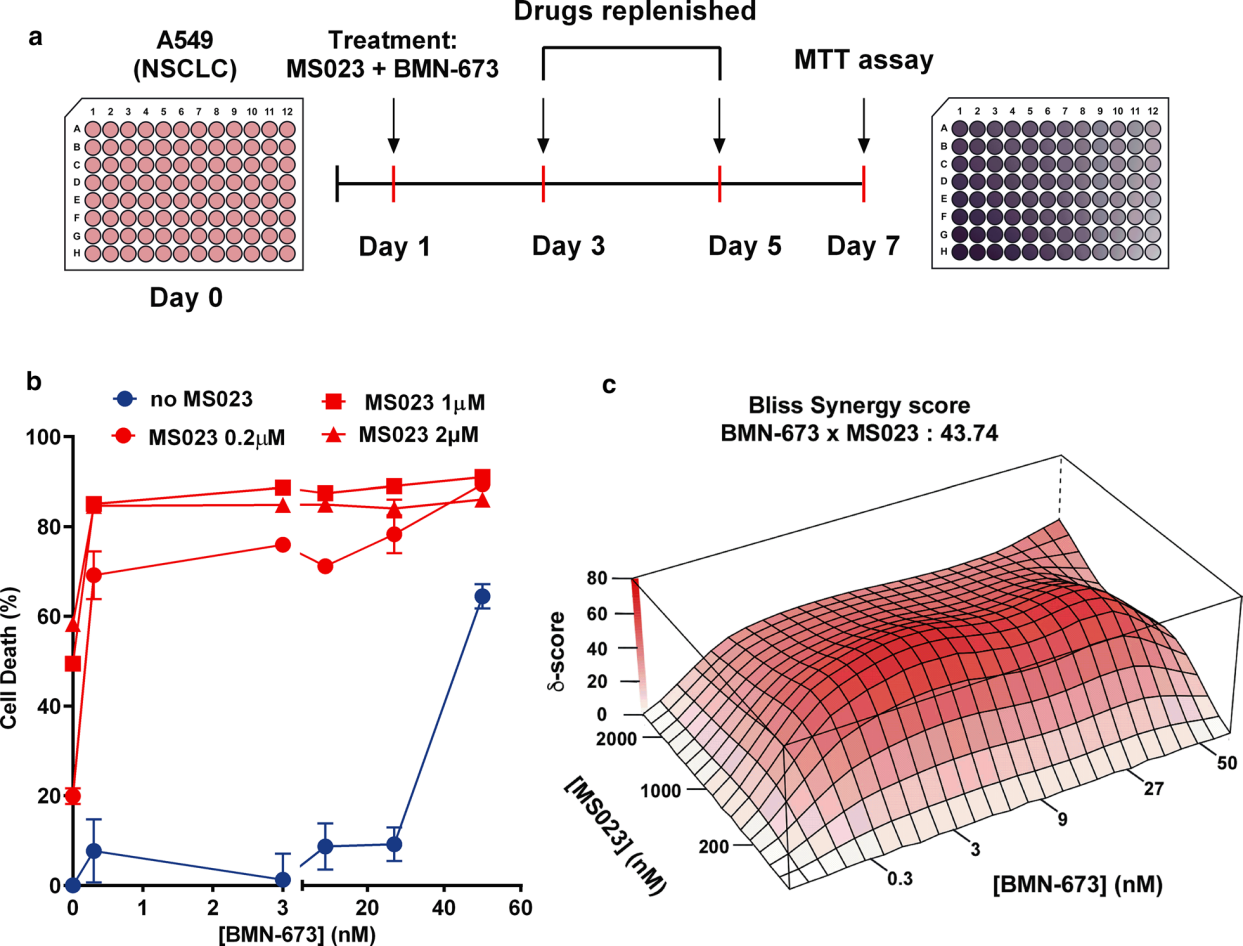
Combination of MS023 with low concentrations of BMN-673 results in synthetic lethality of A549 lung adenocarcinoma cells. **a** Schematic for generating cell viability curves using the MTT assay. **b** Cell viability curves from A549 cells treated with a range of BMN-673 (0.3–60 nM) alone (blue line), or in combination with 0.2, 1, or 2 μM MS023 (red line) (*n* = 3). **c** The open-source R package SynergyFinder was used to visualize the dose–response of the combination of BMN-673 and MS023 in A549 cells and to calculate Bliss synergy scores. The Bliss synergy score is presented on the z-axis of the Bliss graph and is used to determine concentrations at which synergy occurs. Highest Bliss synergy score is highlighted in the graph

**Fig. 4:**
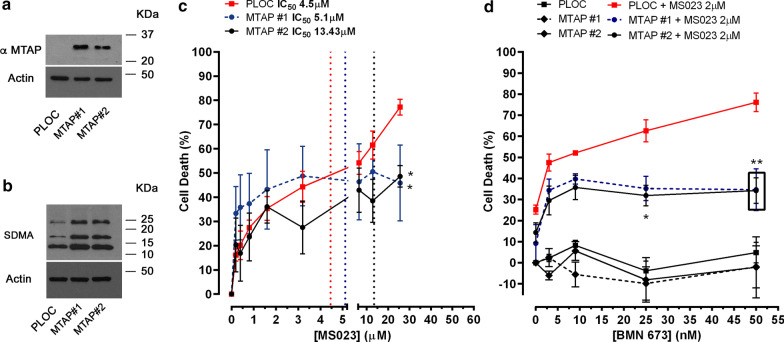
Synergistic effect of MS023 and BMN-673 is dependent on MTAP deficiency in A549 cells. **a** Immunoblotting of A549 cells infected with the empty lentivector (pLoc) or pLoc-MTAP. Clones #1 and #2 show the re-expression of MTAP using anti-MTAP antibodies. Antibodies against β-actin were used to show equivalent loading. The molecular mass markers are shown in kDa. **b** Same as panel A except the cellular lysates were immunoblotted with anti-SDMA and β-actin antibodies as indicated. **c** MTT cell viability assays were performed with A549 (PLOC) and A549 (MTAP #1, #2) treated with a range of MS023 concentrations. Dotted vertical lines represent IC_50_ values (*n* = 3). (*p < 0.05, two-way ANOVA) indicates a statistical difference PLOC and MTAP clones. **d** Cell viability curves as determined by MTT assay of the A549 clones treated with a range of BMN-673 in combination 2 µM MS023 (*n* = 4). **(*p* < 0.01, two-way ANOVA) indicates a statistical difference PLOC and MTAP clones

To confirm the activity of PARPi identified in our screens, we performed MTT-based cell viability assays (Fig. 3a). We used BMN-673 (Talazoparib), one of the recently approved PARPi, which displays high potency by trapping PARP to DNA lesions [34, 35]. We used lower doses of MS023 while increasing treatment duration to 7 days as previously reported to significantly impact A549 cell viability [29]. We found that even at very low concentrations of BMN-673 (0.3 nM), the combination of the two inhibitors significantly killed more than 80% of the cells (Fig. 3b). We calculated the synergy score using the Bliss Synergy method and found that BMN-673 combined with MS023 produced a synergistic effect on cell death (Fig. 3c). These results demonstrate a significant synergistic effect at very low concentrations of PARP and type I PRMT inhibitors. Next, we asked whether the MTAP deficiency in A549 cells could play a role in the synergistic interaction between type I PRMT and PARP inhibitors. A549 cells were stably transfected with an MTAP expression vector and we generated two clones (A549 MTAP #1 and #2), which re-expressed the MTAP protein as visualized by immunoblotting (Fig. 4a). To confirm the restored MTAP enzymatic activity, we measured symmetric arginine dimethylation (SDMA) levels by immunoblotting. MTAP re-expression in A549 clones #1 and #2 had an increase in SDMA levels, consistent with earlier findings that MTAP reduces MTA levels and de-represses PRMT5 inhibition (Fig. 4b) [31–33, 36].

First, we performed MTT assays in control (PLOC) and MTAP-expressing (clones #1, #2) A549 cells (Fig. 4c) to determine their sensitivity to MS023. The presence of MTAP reduced cell death induced by MS023 (PLOC-IC_50_: 4.4 µM; MTAP#1-IC_50_: 5.1 µM; MTAP#2-IC_50_: 13.4 µM). Then, A549 cells were treated with low dose MS023 (2 µM) and various doses of PARPi BNM-673 in the absence (PLOC) or in presence of MTAP (Fig. 4d). Interestingly, the presence of MTAP reduced cell death by at least 50% after exposure to the combination of MS023 and BMN-673 (at 25 and 50 nM), demonstrating that MTAP desensitizes A549 cell to the drug combination. To further explore the potential of the combination, we used two other MTAP-deficient NSCLC cell lines, SK-LU-1 and HC4006. Similarly to A549 cells, MTAP-expression conferred resistance to MS023-induced cell death in both cell lines as shown by increased IC_50_ values (Additional file 1: Figure S2A-D). Interestingly, the combination of MS023 and BMN-673 produced synergistic cell death in SK-LU-1 and HCC4006 (Additional file 1: Figure S2E-H). In contrast to A549 cells, MTAP expression in SK-LU-1 and HC4006 cells did not alter cell death induced by the drug combination, suggesting the involvement of other mechanisms (Additional file 1: Figure S2E, F). It is noteworthy that SK-LU-1 and HCC4006 cells (with or without MTAP) had slower doubling times than A549 cells suggesting that the impact of PARPi is likely to be dependent on the number of cell divisions. Additional experiments need to address these issues in the context of combination PRMTi and PARPi.

**Fig. 5:**
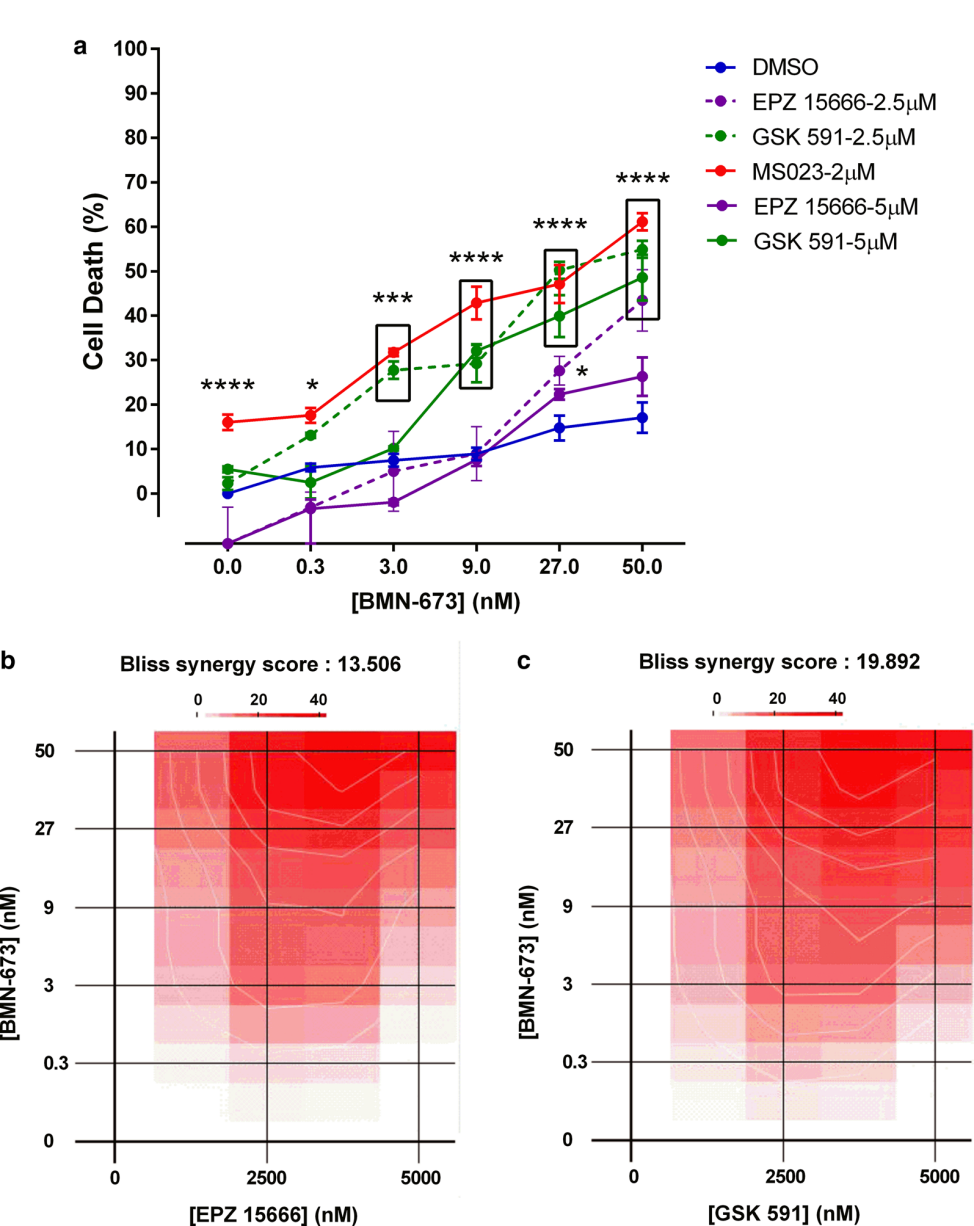
BMN-673 also synergizes with PRMT5 inhibitors. **a** A549 cells were treated for 7 days with DMSO, MS023 or either the PRMT5 inhibitor EPZ015666 or GSK-591 in presence of absence of BMN-673. Cell death was measured using the Viacount reagent (*p < 0.05; ***p < 0.001; ****p < 0.0001; two-way ANOVA). **b** Synergy map showing the interaction between EPZ15666 and BMN-673 in A549 cells. **c** Synergy map showing the interaction between GSK-591 and BMN-673 in A549 cells

Next we asked whether inhibitors of other types of PRMTs, such as PRMT5 (EPZ015666 and GSK591) could synergize with PARPi BMN-673. Cell death was measured in A549 cells using the Viacount reagent after treatment with MS023 (2 µM), EPZ015666 (2.5–5 µM), and GSK591 (2.5–5 µM) alone or in combination with BMN-673 (0.3–50 nM; Fig. 5a). Interestingly, the type I PRMT inhibitor (MS023) demonstrated superiority to induce cell death in combination with the lowest dose of PARPi (0.3 nM), as compared to PRMT5 inhibitors. However, higher doses of PARPi enhanced the activity of both PRMT5 inhibitors (producing synergistic Bliss synergy scores), as also observed by others [20], suggesting some redundancy between PRMT1 and PRMT5 pathways (Fig. 5b, c). Overall, the data support the rationale to combine PRMT inhibitors with PARPi to induce lung cancer cell death at low doses.

### A549 cells treated with a combination of MS023 and BMN-673 accumulate γ-H2AX foci

**Fig. 6:**
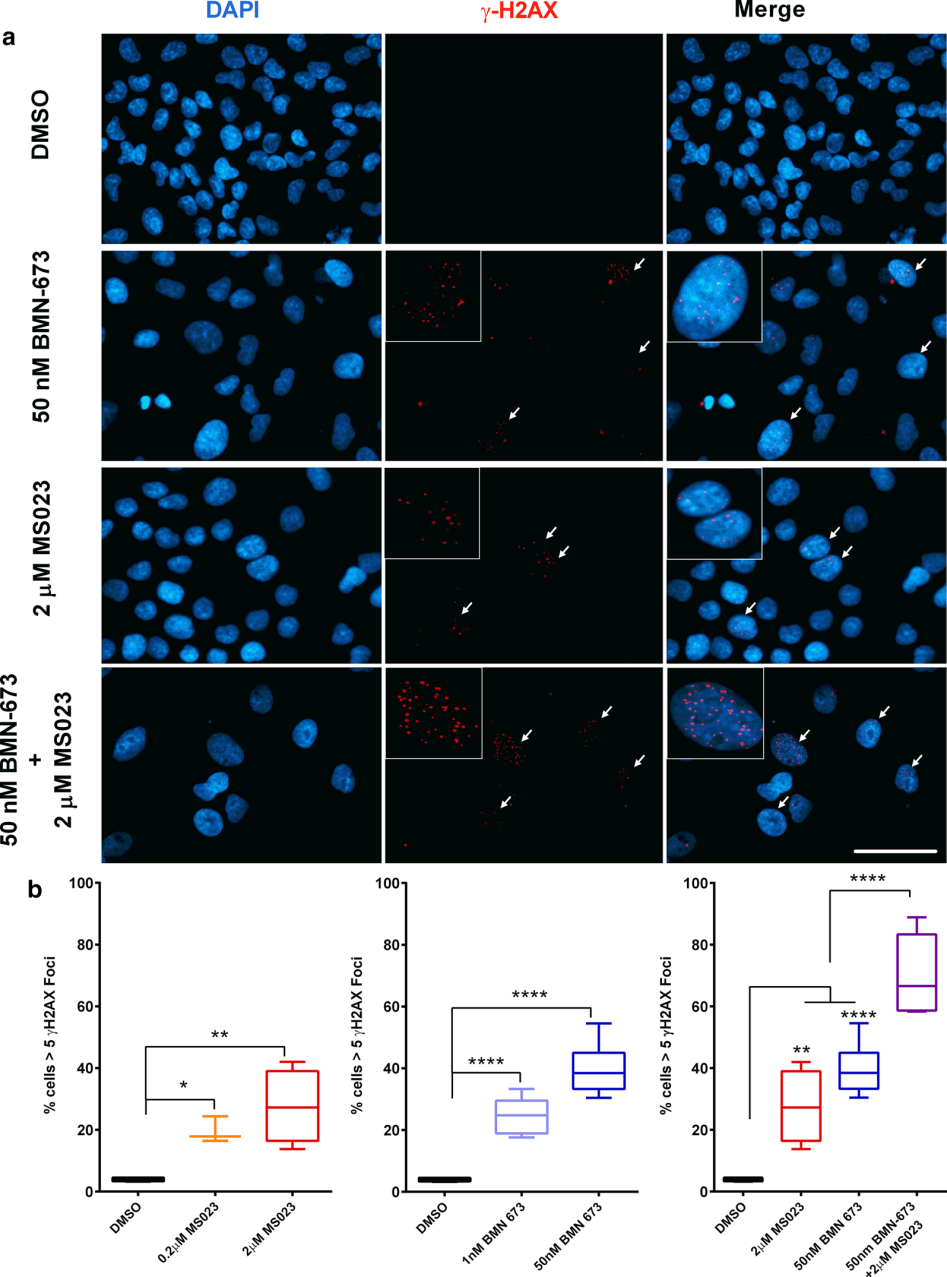
Accumulation of DNA double-strand breaks in A549 cells treated with MS023 and BMN-673. **a** Representative images of A549 cells treated with the indicated drug and concentrations for 7 days and stained for γ-H2AX. Scale bar represents 50 μm in all images. White arrows indicate cells with > 5 γ-H2AX foci. **b** Quantification of γ-H2AX foci in treated A549 cells. Box-and-whisker plots represent the percentage of cells with > 5 γ-H2AX foci, taken from a minimum total of 200 cells in each treatment group. ANOVA was used to compare treatment versus DMSO control, *p* values are presented within the graphs

PARP inhibitors are well-known for generating synthetic lethality in *BRCA*-mutant breast and ovarian cancer cells, which is largely attributed to a deficiency in homologous recombination (HR) [37–40]. Considering that both PRMTs and PARPs are functionally involved in the DNA damage response, we performed γ-H2AX foci analysis to monitor DNA damage in A549 cells. Treatment for 7 days with either MS023 (2 µM) or BMN-673 (50 nM) induced γ-H2AX foci formation, as observed by immunofluorescence (Fig. 6a). These observations suggest that the single drug treatments were able to induce a certain level of DNA damage on their own. The combination therapy using both inhibitors led to a significant increase in γ-H2AX foci, implying increased DNA damage being responsible for the reduced viability (Fig. 6a). More precisely, low concentrations of BMN-673 (1–50 nM) produced a significant increase in the percentage of cells (1 nM, 24.8%; 50 nM, 40.4%) with greater than five γ-H2AX (*p* < 0.0001), as compared to untreated cells (3.9%; Fig. 6b). Low concentrations of MS023 (0.2–2 µM) produced similar effects (0.2 µM, 19.6%; 2.0 µM, 27.6%; Fig. 6b). The combination of BMN-673 (50 nM) and MS023 (2 µM) produced a significant increase in the percentage of cells (69.5%) with greater than five γ-H2AX foci (*p* < 0.001; Fig. 6b). Overall, the data show that the drug combination of PARP and type I PRMT inhibitors elevate cytotoxicity by augmenting DNA damage in MTAP-negative A549 cells.

### PRMT inhibitors restore PARP inhibitor sensitivity

**Fig. 7:**
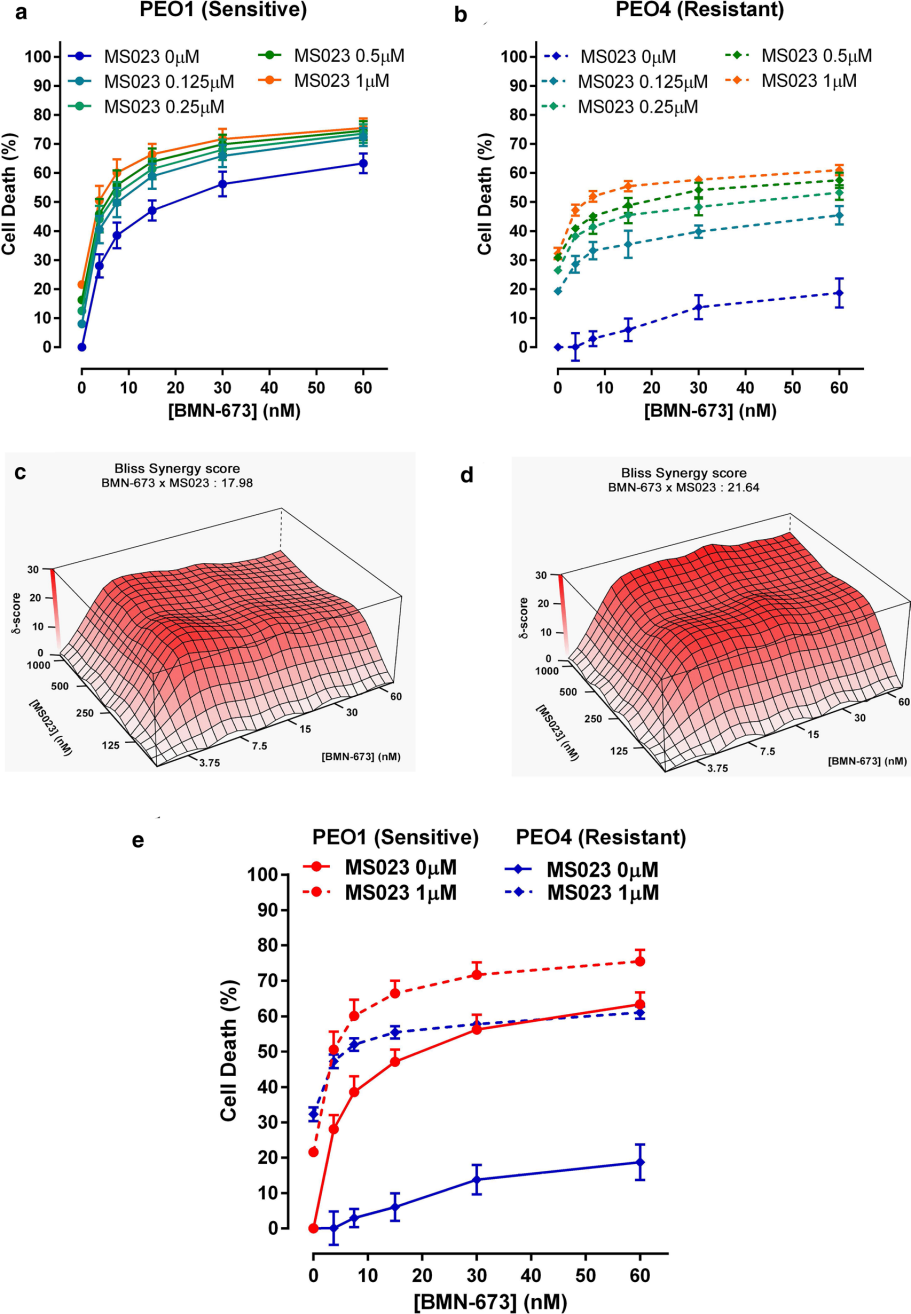
Treatment with MS023 renders PARPi-resistant PEO4 ovarian adenocarcinoma cells sensitive to BMN-673. **a, b** Cell viability curves from PEO1 (**a**) and PEO4 (**b**) cells treated with a range of BMN-673 (3.75–60 nM) alone, or in combination with 0.125, 0.25, 0.5, or 1 µM MS023. **c, d** The open-source R package SynergyFinder was used to visualize the dose–response of the combination of BMN-673 and MS023 in PEO1 (**c**) and PEO4 (**d**) cells and to calculate Bliss synergy scores. The Bliss synergy score is presented on the *z*-axis of the Bliss graph and is used to determine concentrations at which synergy occurs. **e** Cell viability curves from PEO1 (red) and PEO4 (blue) cells treated with a range of BMN-673 (3.75–60 nM) alone, or in combination with 1 µM MS023

We asked whether the synergic relationship between MS023 and BMN-673 could be extended to PARPi-resistant cells. We used the ovarian cancer cell lines PEO1 and PEO4, which are derived from the same patient [41]. PEO1 cells are BRCA2-deficient and show sensitivity to PARPi, MS023 and their combination (Fig. 7a). PEO4 cells have a secondary BRCA2 mutation, which restores BRCA2 expression and are therefore resistant to BMN-673 alone (Fig. 7b). Interestingly, we observed a synergistic effect of the combination of MS023 and BMN-673 not only on the PEO1 cells, but also in the PEO4 BMN-673-resistant cells, as demonstrated by the high bliss score values in both cell lines (Fig. 7c, d). These results indicate that type I PRMT inhibition in combination with PARPi may be used to treat tumors that present resistance to PARPi through HR restoration (Fig. 7e).

## Discussion

In the present manuscript, we performed a chemical screen to identify epigenetic and anticancer drugs that synergize with a type I PRMT inhibitor, MS023. Homozygous deletion of the *MTAP* gene is frequently (~ 40%) seen in lung cancer patients [30], and leads to elevated levels of MTA, a metabolite known to act as an endogenous inhibitor of PRMT5 activity [31–33]. As such, *MTAP*-deficient cancer cells are inherently sensitive to inhibition of PRMT1 [24, 28, 29]. As resistance occurs frequently in NSCLCs, we aimed to identify additional compounds that could function in combination therapy with MS023 to significantly increase its cytotoxicity in the *MTAP*-negative A549 cell line. We identified several PARPi that had a strong synergy index with type I PRMT inhibition. We further examined the combination of MS023 and the PARP inhibitor BMN-673 (Talazoparib), and observed strong synergistic interaction at low nM concentrations in *MTAP-*negative A549, SK-LU-1 and HCC4006 NSCLC cells. The re-introduction of MTAP decreased the sensitivity of the combination therapy in A549 cells. Importantly, PARP inhibitor sensitive and resistant cells (PEO1, PEO4) were both sensitive to the combination therapy of MS023 and BMN-673. These data suggest that type I PRMT inhibitors may have a wide therapeutic window targeting certain NSCLC and ovarian cancers in combination with PARP inhibitors.

PARPs are a family of enzymes with at least 18 members that catalyze the addition of poly(ADP-ribose) to various biological molecules. The most well-studied member of the PARP family, PARP1, plays an important role in DNA damage repair and is able to catalyze poly (ADP-ribose) chains [42, 43]. Inhibitors of PARP generate synthetic lethality in *BRCA1*- and *BRCA2*-mutant breast/ovarian cancer cells [37–40]. Four PARP inhibitors have been approved by regulatory agencies, including Olaparib and Rucaparib in *BRCA*-mutated ovarian cancer; Niraparib in epithelial ovarian, fallopian tube and primary peritoneal cancer; and Talazoparib in *BRCA*-mutated breast cancer [44, 45]. Importantly, several compounds are being pursued as promising combinatorial agents with PARPi in several types of cancers [46–49]. Defining additional inhibitors that will work in combination with PARPi is paramount since resistance to PARPi single agent can occur through increased drug efflux, reactivation of homologous recombination, restoration of replication fork stability, or loss of DNA double-strand break resection inhibition [50].

We now identify type I PRMT inhibitors as functioning to kill NSCLC. Type I PRMT inhibition or PRMT1 deficiency is known cause DNA damage with homologous recombination defects [18]. Thus, it is likely that MS023 creates HR defects similar to what is observed in *BRCA*-mutated cancers, thereby creating a vulnerability for PARPi. The combination of MS023 and PARP inhibitors can be used to ablate HR-proficient cancers. Indeed, PARP inhibitors have also been used in combination with other agents to treat HR-proficient cancers [44, 51–53].

At sites of DNA damage, negatively charged PARylated proteins including PARP1 itself may recruit positively charged RGG/RG motif-containing methylated proteins. Arginine methylation plays a key role in the DNA damage response (DDR), and is known to occur at the RGG/RG motifs on several DDR proteins, including MRE11, 53BP1, and BRCA1 [21, 54–57]. Therefore, arginine methylation of these proteins may affect their interactions with chains of PAR. Collectively, the combination of lack of arginine methylation and PARylation leads to DNA repair defects, causing synthetic lethality in *MTAP*-negative NSCLC.

## Conclusions

As with any chemotherapy, the potential cytotoxicity of PARPi either alone or in combination with other agents needs to be considered. Importantly, we show that low concentrations of BMN-673 PARPi (0.3 nM) were sufficient to kill lung cancer cells in combination with MS023. As demonstrated in our present study, the PARP and PRMT combination may be useful for recombination (HR) repair-deficient and proficient cancers. Due to the redundancy of PRMT1 and PRMT5 pathways, we also demonstrated that PARPi were effective in combination with PRMT5 inhibitors. Indeed, PARPi have been shown to synergize with PRMT5 inhibitors [20]. In sum, our findings show that targeting PRMTs in combination with PARP inhibitors presents as a new therapy option for NSCLC cancers that are HR-proficient.

## Materials and methods

### Cell culture and generation of stable cell lines

Lung carcinoma cells A549 (ATCC CCL-185), SK-LU-1 (ATCC HTB57) and HCC4006 (ATCC CRL-2871) were cultured in F12K, DEMEM and RPMI-1640 (GE Healthcare Life Sciences, Canada) respectively, supplemented with 10% of FBS (Wisent, Canada), and maintained in a humidified incubator with 5% CO2 at 37 °C. Cells were regularly checked for mycoplasma infection and kept at low passages. Cell lines re-expressing MTAP were generated by infecting the *MTAP*-deficient cell lines A549, SK-LU-1 and HCC4006 with human MTAP lentivirus (pLoc-MTAP). The MTAP-infected cells were treated with 3 µg/ml blasticidin and single clones were selected. MTAP expression was confirmed by Western blot using anti-MTAP antibody (Cell Signaling Technology, 4158). As a control, the cells were also infected with lentiviral empty vector (pLoc) and a pool of blasticidin-resistant cells were selected. Ovarian adenocarcinoma cells PEO1 and PEO4 were a kind gift from Scott H. Kaufmann (Mayo Clinic). Cells were cultured in OSE (Wisent, Canada) supplemented with 10% of FBS (Wisent, Canada), and maintained in a humidified incubator with 5% CO_2_ at 37 °C. For combination survival assays, PEO1 and PEO4 cells were seeded at 3,000 cells/well in flat bottom black 96-well plates (Corning). One day after plating, cells were treated with the indicated concentrations of MS023 (Cayman Chemical) and/or BMN-673 (SelleckChem, S7048), or an equivalent concentration of vehicle (DMSO, Sigma-Aldrich). Media containing inhibitors or DMSO were replenished every 48 h. Six days after the first treatment, Hoechst 33342 (Thermo Fischer Scientific, H3570) was added at a final concentration of 10 µg/ml and the plates were returned to the incubator for 30 min. The wells were imaged using a Cytation5 plate imager (BioTek) and the nuclei counted using Gen5 software. To normalize the results, the number of nuclei in the inhibitor-treated wells was divided by the number of nuclei of the DMSO-treated wells.

### Epigenetic drug screen and synergy index calculation

For screening purposes, A549 cells were seeded at 20 K cells/well in flat-bottom 96-well plates (Sarstedt). One day after, cells were treated with 16.5 μM MS023 (Cayman Chemical) or an equivalent concentration of vehicle (DMSO, Sigma-Aldrich). The next day, 181 drugs from the SelleckChem epigenetic library (Epigenetics Compound Library (96-well)-Z203065-100 μl-L1900) were added at 10 μM for 24 h. Each plate contained controls to assess the individual effects of DMSO and MS023 on cells. On the third day, cell viability was measured using Viacount (Luminex, 4000-0040) on Guava flow cytometer (Millipore) as follows. Media was collected for each well and kept aside for later, cells were rinsed in 200 μl PBS without calcium (Wisent) and incubated with 0.25% trypsin (Gibco) for 5 min at 37 °C. Media of each well was added to stop trypsinization and mixed thoroughly with Integra 96-well automated pipettor. One control well (DMSO) was heat-killed to obtain a total cell death control. Viacount solution (25 µl) was added and mixed to each well, as suggested per manufacturer guidelines. After incubation (5 min at room temperature, in the dark), cell viability was analyzed on the Guava flow cytometer. The drug library was screened in monotherapy, as previously described. The Synergy Index (SI) is calculated as follows: SI = (Viability in monotherapy × MS023 Viability effect)/Viability in combination. We also calculated a synergy/antagonism percentage value to associate a score to the combination as compared to monotherapy. The synergy/antagonism percentage is calculated as follows (1 −(1/Synergy Index))*100.

Enrichment score was calculated to express relative effect of a drug class to other drug classes present in the library. Enrichment score is the ratio of its enrichment index and the global index of the whole screen. To calculate the enrichment index, we created a frequency table of each drug response per group. The matrix is composed of 21 bins from -100 to 100, with a step of 10. Then, we calculate the representative percentage (weight) of each bin per group (bin weight = bin frequency / total number of compounds per group), to create 21 pairs per group. Finally, we sum up for each group the product of each (bin, weight) pairs. The global index is calculated the same way. The enrichment score corresponds to the ratio of each group index divided by the global index: a score > 1 shows an enrichment whereas a score < 1 shows the opposite.

### MTT assay

A549, SK-LU-1 and HCC4006 stable cells infected with MTAP or empty lentiviral vectors were treated as described above with different dosage of inhibitors for 7 days. For treatment, the type I PRMT inhibitor MS023 was dissolved in DMSO to prepare a 3 mM stock solution, and cells were treated with a final concentration of 0.2–2 μM as indicated. The PARP1/2 inhibitor BMN-673 (SelleckChem, S7048) was also dissolved in DMSO to prepare a 3 mM stock solution, and cells were treated with a final concentration of 0.3–50 nM as indicated. For treatment, cells were seeded on day zero and inhibitors or DMSO were added after 16 h. Media and inhibitors or DMSO were replenished every 48 h. Cell viability was assessed using the MTT assay kit (Abcam, ab211091) according to manufacturer's instructions. Briefly, cells were grown in a 96-well plate and each treatment group was repeated in triplicate. On the 7th day, media was carefully aspirated and 100 μl of 1X MTT reagent was added to each well, and the plate was incubated for 3 h at 37 °C. Following incubation, 150 μl of MTT solvent was added to each well and incubated at room temperature on an orbital shaker for 15 min prior to reading absorbance at OD = 590 nm. To normalize absorbance values, each value for the inhibitor-treated wells was divided by the absorbance of the DMSO-treated wells. Absorbance values are proportional to cell number, so percent cell death was determined by subtracting the normalized values from 1. An evaluation of the drug combination effect was carried out using the Bliss Independence dose–response calculation [58, 59].

### Immunofluorescence

A549 cells were cultured on glass coverslips under the cell culture treatment conditions described above for the MTT assay. On day 7, coverslips were transferred to a solution of 4% paraformaldehyde in PBS and fixed for 15 min at room temperature. Cells were then permeabilized with 0.2% Triton X-100, 0.125 M glycine in PBS for 12 min at room temperature. Blocking followed for 1 h at room temperature with 2% BSA, 2% horse serum and 0.1% Triton X-100 in PBS. γ-H2AX foci were detected using anti-gamma H2A.X (Abcam, ab11174) diluted 1:1000 in blocking buffer and incubated at 4 °C for 16 h. Cells were washed three times with PBS for 10 min and incubated with secondary antibody (AlexaFluor anti-mouse 488 nm) diluted 1:400 for 45 min in the dark at room temperature. Cells were then washed three times for 10 min with PBS. Finally, coverslips were inverted and mounted onto a microscope slide with Immu-Mount (Fisher Scientific) and DAPI for counterstain. Slides were imaged on a Zeiss Axio Imager M1 microscope (Carl Zeiss, Thornwood NY), and resulting images were analyzed using Zeiss' ZEN Digital imaging suite software. A minimum of 200 cells per treatment condition were imaged, and cells with > 5 γH2AX foci were quantified and divided by total number of cells as determined by DAPI counterstain.

### Statistical analyses

Statistical significance was determined using GraphPad Prism version 6.0 software to perform unpaired t tests, where *p* values less than 0.05 were accepted as significant.

## Supplementary Information

**Additional file 1**. **Figure S1. Validation of the drug screen. **Graphic showing original data from the screen (plain circle) highest (20) and lowest (20) hits. Results from the validation are labelled with (+) if validation was confirmed or (-) if validation failed. Pie chart summarizes result of validation process; 28 of 40 compounds matched the primary output, leading to a validation rate for the screen of 70%. **Figure S2. MS023 and BMN-673 synergy dependency on MTAP in NSCLC cell lines. A)** Immunoblotting of SK-LU-1 and HCC4006 cell lines infected with the empty lentivector (pLoc) or pLoc-MTAP. Clones #1 and #2 show the re-expression of MTAP using anti-MTAP antibodies. Antibodies against β-actin were used to show equivalent loading. The molecular mass markers are shown in kDa. **B)** Same as panel A except the cellular lysates were immunoblotted with anti-SDMA and β-actin antibodies as indicated. **C-D)** Cell death curves as determined by MTT assay of the SK-LU-1 and HC4006 clones treated with a range of MS023 concentrations. Dotted vertical lines represent IC_50_ values for each cell line (SK-LU-1: n=5; HCC4006: n=4). Stars (*: *p* <0.05; **: *p* <0.01; ***: *p* <0.001, ****: *p* <0.0001; two-way ANOVA). **E-F)** Cell death curves as determined by MTT assay of the SK-LU-1 and HCC4006 clones treated with a range of BMN-673 in combination 10 µM MS023 (SK-LU-1, n=4) or 0.2 µM MS023 (HCC4006, n=6). **G-H)** Bliss synergy scores calculated for BMN-673 and MS023 combination treatment in SK-LU-1 and HCC4006 cells, respectively. **Table 1.** Drug screening results. Synergy indexes are shown for the 181 compounds treated in combination with MS023 in A549 cells. **Table 2**. Drug validation results. Synergy indexes are shown for the 40 compounds (20 highest and 20 lowest hits) treated in combination with MS023 in A549 cells. Validation and screening synergy indexes are shown. Cells colored in grey highlight opposing results between screen and validation results. Overall, the validation rate was at 70% (28/40).

## Data Availability

All data generated or analysed during this study are included in this published article and its supplementary information files.

## Abbreviations

aDMA: Asymmetric dimethylarginine
DNMT: DNA methyltransferase
DSB: Double-strand break
HDAC: Histone deacetylase
MMA: Monomethylarginine
MTA: Methylthioadenosine
MTAP: Methylthioadenosine phosphorylase
NSCLC: Non-small cell lung carcinoma
PARP: Poly(ADP)–ribose polymerase
PARPi: PARP inhibitor
PRMT: Protein arginine methyltransferase
SAH: S-adenosylhomocysteine
SAM: S-adenosylmethionine
sDMA: Symmetric dimethylarginine
