# Supplementary material for: Synergistic effects of type I PRMT and PARP inhibitors against non-small cell lung cancer cells
Source: Clin Epigenetics. 2021 Mar 10;13:54. doi: 10.1186/s13148-021-01037-1 (PMC7948358; doi:10.1186/s13148-021-01037-1)
Supplement: Supplementary file 1 — Additional file 1. Figure S1. Validation of the drug screen. Graphic showing original data from the screen (plain circle) highest (20) and lowest (20) hits. Results from the validation are labelled with (+) if validation was confirmed or (-) if validation failed. Pie chart summarizes result of validation process; 28 of 40 compounds matched the primary output, leading to a validation rate for the screen of 70%. Figure S2. MS023 and BMN-673 synergy dependency on MTAP in NSCLC cell lines. A) Immunoblotting of SK-LU-1 and HCC4006 cell lines infected with the empty lentivector (pLoc) or pLoc-MTAP. Clones #1 and #2 show the re-expression of MTAP using anti-MTAP antibodies. Antibodies against β-actin were used to show equivalent loading. The molecular mass markers are shown in kDa. B) Same as panel A except the cellular lysates were immunoblotted with anti-SDMA and β-actin antibodies as indicated. C-D) Cell death curves as determined by MTT assay of the SK-LU-1 and HC4006 clones treated with a range of MS023 concentrations. Dotted vertical lines represent IC50 values for each cell line (SK-LU-1: n=5; HCC4006: n=4). Stars (*: p <0.05; **: p <0.01; ***: p <0.001, ****: p <0.0001; two-way ANOVA). E-F) Cell death curves as determined by MTT assay of the SK-LU-1 and HCC4006 clones treated with a range of BMN-673 in combination 10 µM MS023 (SK-LU-1, n=4) or 0.2 µM MS023 (HCC4006, n=6). G-H) Bliss synergy scores calculated for BMN-673 and MS023 combination treatment in SK-LU-1 and HCC4006 cells, respectively. Table 1. Drug screening results. Synergy indexes are shown for the 181 compounds treated in combination with MS023 in A549 cells. Table 2. Drug validation results. Synergy indexes are shown for the 40 compounds (20 highest and 20 lowest hits) treated in combination with MS023 in A549 cells. Validation and screening synergy indexes are shown. Cells colored in grey highlight opposing results between screen and validation results. Overall, the validation rate was at 70% (28/40) [file 13148_2021_1037_MOESM1_ESM.pdf]

Supplemental Figure 1

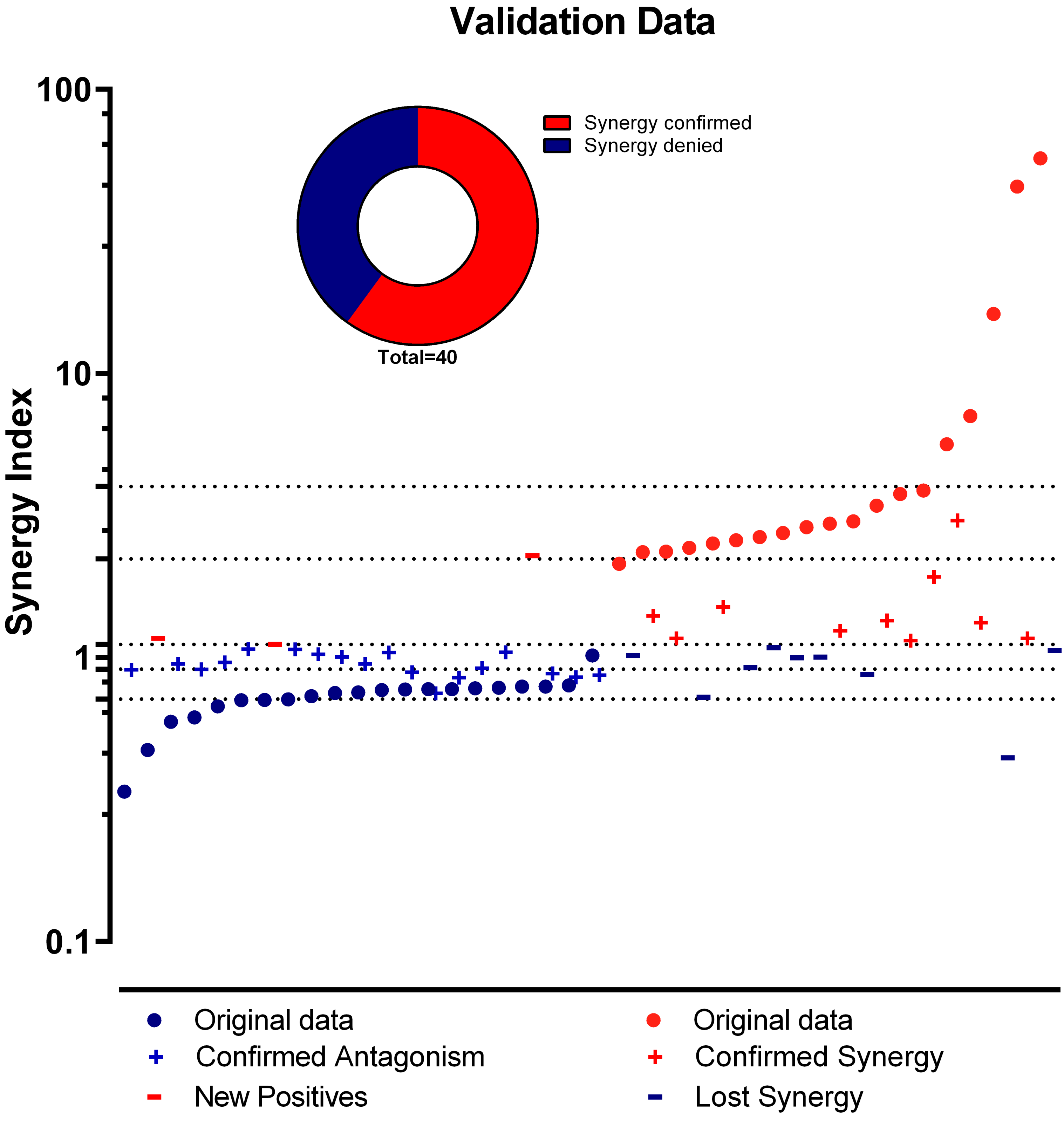

Supplemental Figure 2

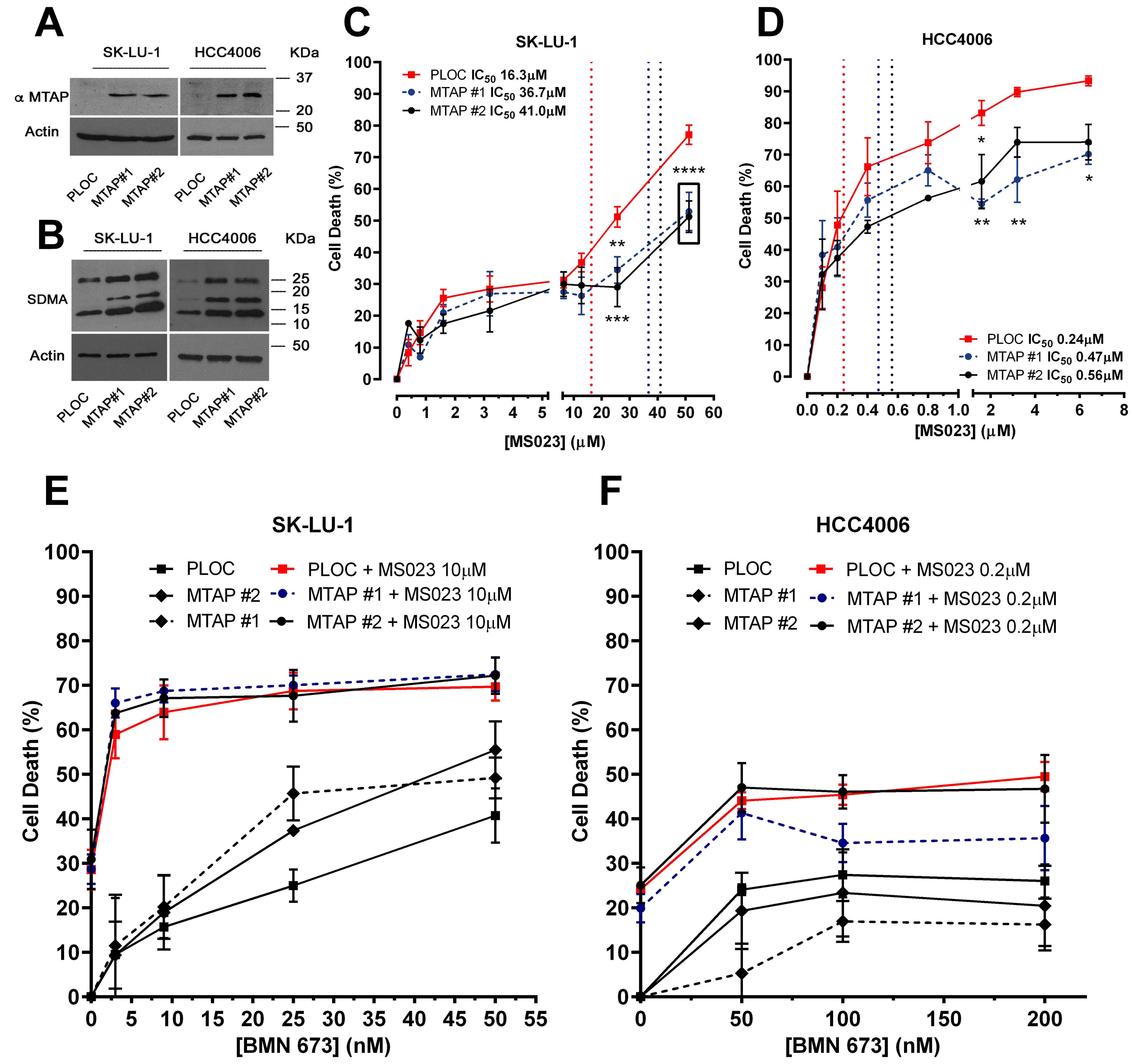

G

BMN 673 (nM)	3	9	25	50
PLOC	20.31	19.68	18.63	13.79
MTAP#1	21.19	17.77	11.19	4.13
MTAP#2	18.52	16.10	11.13	2.70

H

BMN 673 (nM)	25	50	100	200
PLOC	-0.12	-4.49	2.759	7.405
MTAP#1	21.05	13.25	0.703	-2.1
MTAP#2	11.07	10.74	7.7	6.284

Supplemental Table 1

Rank	Name	Target	Synergy Index
1	AMG-900	Aurora Kinase	0.34
2	TAK-901	Aurora Kinase	0.47
3	Gandotinib (LY2784544)	JAK	0.59
4	IOX2	HIF	0.61
5	AZD1480	JAK	0.67
6	Sirtinol	Sirtuin	0.71
7	Abexinostat (PCI-24781)	HDAC	0.71
8	2-Methoxyestradiol (2-MeOE2)	HIF	0.71
9	Aurora A Inhibitor I	Aurora Kinase	0.73
10	RG108	DNA Methyltransferase,Transferase	0.75
11	GSK-LSD1 2HCl	Histone Demethylase	0.75
12	Valproic acid	HDAC	0.77
13	Norfloxacin	Topoisomerase	0.77
14	Ruxolitinib (INCB018424)	JAK	0.77
15	Pirarubicin	Topoisomerase	0.77
16	Selisistat (EX 527)	Sirtuin	0.78
17	Droxinostat	HDAC	0.78
18	AR-42	HDAC	0.79
19	Tubastatin A HCl	HDAC	0.79
20	GSK2879552 2HCl	Histone Demethylase	0.8
21	Quisinostat (JNJ-26481585) 2HCl	HDAC	0.8
22	Tofacitinib (CP-690550;Tasocitinib)	JAK	0.81
23	MS436	Epigenetic Reader Domain	0.82
24	GSK591	Histone Methyltransferase	0.83
25	PCI-34051	HDAC	0.83
26	WHI-P154	JAK	0.83
27	PJ34	PARP	0.83
28	MS023	Histone Methyltransferase	0.83
29	EPZ015666(GSK3235025)	Histone Methyltransferase	0.84
30	BRD4770	Histone Methyltransferase	0.84
31	Trichostatin A (TSA)	HDAC	0.84
32	PHA-680632	Aurora Kinase	0.84
33	Resveratrol	Autophagy,Sirtuin	0.84
34	Anacardic Acid	Histone Acetyltransferase	0.84
35	AZ 960	JAK	0.85
36	KW-2449	FLT3	0.85
37	GSK J1	Histone Demethylase	0.86
38	UPF 1069	PARP	0.86
39	NVP-BSK805 2HCl	JAK	0.86
40	I-BET-762	Epigenetic Reader Domain	0.86
41	Bromosporine	Epigenetic Reader Domain	0.87
42	AZD1208	Pim	0.87
43	RVX-208	Epigenetic Reader Domain	0.87
44	Vorinostat (SAHA; MK0683)	HDAC	0.87
45	MK-5108 (VX-689)	Aurora Kinase	0.88
46	Mirin	ATM/ATR	0.88
47	ORY-1001 (RG-6016) 2HCl	Histone Demethylase	0.88
48	ML324	Histone Demethylase	0.88
49	MG149	Histone Acetyltransferase	0.89
50	EPZ011989	Histone Methyltransferase	0.9
51	Ellagic acid	Topoisomerase	0.9
52	Remodelin	Histone Acetyltransferase	0.9
53	UNC0631	Histone Methyltransferase	0.9
54	CUDC-101	HDAC	0.9
55	GSK503	Histone Methyltransferase	0.91
56	NVP-TNKS656	PARP	0.93
57	Entacapone	Histone Methyltransferase	0.93
58	Ricolinostat (ACY-1215)	HDAC	0.93
59	Nexturastat A	HDAC	0.93

60	BIX 01294	Histone Methyltransferase	0.94
61	Decitabine	DNA Methyltransferase	0.94
62	SGC-CBP30	Epigenetic Reader Domain	0.96
63	ZM 39923 HCl	JAK	0.96
64	Barasertib (AZD1152-HQPA)	Aurora Kinase	0.96
65	Sodium Phenylbutyrate	HDAC	0.96
66	INO-1001 (3-Aminobenzamide)	PARP	0.97
67	Daphnetin	PKA,EGFR,PKC	0.98
68	Clevudine	DNA/RNA Synthesis	0.99
69	CYC116	Aurora Kinase	0.99
70	A-366	Histone Methyltransferase	0.99
71	AG-14361	PARP	0.99
72	PFI-1 (PF-6405761)	Epigenetic Reader Domain	1
73	MI-3 (Menin-MLL Inhibitor)	Histone Methyltransferase	1
74	CPI-360	Histone Methyltransferase	1.01
75	Alisertib (MLN8237)	Aurora Kinase	1.01
76	Decernotinib (VX-509)	JAK	1.01
77	Pracinostat (SB939)	HDAC	1.01
78	ENMD-2076 L-(+)-Tartaric acid	Aurora Kinase	1.02
79	SMI-4a	Pim	1.02
80	PFI-3	Epigenetic Reader Domain	1.02
81	AT9283	Aurora Kinase	1.02
82	SNS-314 Mesylate	Aurora Kinase	1.03
83	UNC669	Epigenetic Reader Domain	1.03
84	OF-1	Epigenetic Reader Domain	1.03
85	TMP269	HDAC	1.03
86	Nedaplatin	DNA/RNA Synthesis	1.04
87	OG-L002	Histone Demethylase	1.04
88	I-BRD9	Epigenetic Reader Domain	1.04
89	RG2833 (RGFP109)	HDAC	1.04
90	Procainamide HCl	DNA Methyltransferase	1.05
91	Lomeguatrib	DNA Methyltransferase	1.05
92	GSK1324726A (I-BET726)	Epigenetic Reader Domain	1.05
93	Tranylcypromine (2-PCPA) HCl	MAO	1.06
94	Momelotinib (CYT387)	JAK	1.06
95	Tubastatin A	HDAC	1.08
96	S-Ruxolitinib (INCB018424)	JAK	1.08
97	Resminostat	HDAC	1.09
98	WP1066	JAK	1.09
99	(+)-JQ1	Epigenetic Reader Domain	1.09
100	Blasticidin S HCl	DNA/RNA Synthesis	1.09
101	BI-7273	Epigenetic Reader Domain	1.09
102	3-deazaneplanocin A (DZNeP) HCl	Histone Methyltransferase	1.1
103	M344	HDAC	1.1
104	Tacedinaline (CI994)	HDAC	1.1
105	GSK J4 HCl	Histone Demethylase	1.1
106	AZ6102	PARP	1.11
107	Niraparib (MK-4827) tosylate	PARP	1.13
108	Quercetin	Src,Sirtuin,PKC,PI3K	1.14
109	Mocetinostat (MGCD0103)	HDAC	1.14
110	Zebularine	DNA Methyltransferase	1.16
111	Baricitinib (LY3009104; INCB028050)	JAK	1.16
112	GSK2801	Epigenetic Reader Domain	1.17
113	Iniparib (BSI-201)	PARP	1.18
114	Daptomycin	DNA/RNA Synthesis	1.19
115	JNJ-7706621	Aurora Kinase	1.2
116	Pacritinib (SB1518)	FLT3	1.2
117	SGC 0946	Histone Methyltransferase	1.21
118	Fedratinib (SAR302503; TG101348)	JAK	1.22
119	CPI-203	Epigenetic Reader Domain	1.23
120	SGI-1776 free base	Pim	1.24

121	MI-2 (Menin-MLL Inhibitor)	Histone Methyltransferase	1.24
122	Ofloxacin	Topoisomerase	1.24
123	GSK1070916	Aurora Kinase	1.24
124	PJ34 HCl	PARP	1.26
125	Oclacitinib	JAK	1.27
126	UNC1215	Epigenetic Reader Domain	1.27
127	Azacitidine	DNA Methyltransferase	1.27
128	CEP-33779	JAK	1.27
129	CPI-169	Histone Methyltransferase	1.28
130	C646	Histone Acetyltransferase	1.29
131	Rucaparib	PARP	1.31
132	MM-102	Histone Methyltransferase	1.32
133	ITSA-1 (ITSA1)	HDAC	1.35
134	CUDC-907	HDAC	1.36
135	Mitomycin C	DNA/RNA Synthesis	1.36
136	OTX015	Epigenetic Reader Domain	1.37
137	JIB-04	Histone Demethylase	1.41
138	PF-CBP1 HCl	Epigenetic Reader Domain	1.43
139	4SC-202	HDAC	1.44
140	EI1	Histone Methyltransferase	1.46
141	Scriptaid	HDAC	1.46
142	AG-490 (Tyrphostin B42)	JAK	1.47
143	SGC707	Histone Methyltransferase	1.48
144	Givinostat (ITF2357)	HDAC	1.48
145	Cytarabine	DNA/RNA Synthesis	1.48
146	RGFP966	HDAC	1.51
147	Danusertib (PHA-739358)	Aurora Kinase	1.51
148	ZM 447439	Aurora Kinase	1.56
149	MLN8054	Aurora Kinase	1.57
150	Pinometostat (EPZ5676)	Histone Methyltransferase	1.61
151	APTSTAT3-9R	STAT	1.63
152	HLCL-61 HCL	Histone Methyltransferase	1.66
153	Carboplatin	DNA/RNA Synthesis	1.67
154	PFI-2 HCl	Histone Methyltransferase	1.71
155	XL019	JAK	1.79
156	Belinostat (PXD101)	HDAC	1.84
157	Roxadustat (FG-4592)	HIF	1.88
158	Olaparib (AZD2281; Ku-0059436)	PARP	1.93
159	ME0328	PARP	2.01
160	Hesperadin	Aurora Kinase	2.07
161	TG101209	FLT3	2.08
162	Tozasertib (VX-680; MK-0457)	Aurora Kinase	2.13
163	ENMD-2076	FLT3	2.14
164	Gemcitabine HCl	DNA/RNA Synthesis	2.35
165	EPZ004777	Histone Methyltransferase	2.36
166	CX-6258 HCl	Pim	2.43
167	UNC0379	Histone Methyltransferase	2.52
168	Tofacitinib (CP-690550) Citrate	JAK	2.59
169	Mizoribine	DNA/RNA Synthesis	2.65
170	IOX1	Histone Demethylase	2.74
171	Procarbazine HCl	DNA/RNA Synthesis	2.87
172	SGI-1027	DNA Methyltransferase	2.96
173	AZD2461	PARP	3.02
174	Panobinostat (LBH589)	HDAC	3.42
175	Entinostat (MS-275)	HDAC	3.76
176	Dacinostat (LAQ824)	HDAC	3.87
177	MC1568	HDAC	5.63
178	SP2509	Histone Demethylase	7.08
179	SRT1720 HCl	Sirtuin	16.18
180	Veliparib (ABT-888)	PARP	45.5
181	Filgotinib (GLPG0634)	JAK	57.18

Supplemental table 2

		Synergy Index	
Name	Target	Screen	Validation
AMG-900	Aurora Kinase	0.34	0.90
TAK-901	Aurora Kinase	0.47	1.17
Gandotinib (LY2784544)	JAK	0.59	0.95
IOX2	HIF	0.61	0.91
AZD1480	JAK	0.67	0.96
Sirtinol	Sirtuin	0.71	1.07
Abexinostat (PCI-24781)	HDAC	0.71	1.11
2-Methoxyestradiol (2-MeOE2)	HIF	0.71	1.07
Aurora Inhibitor I	Aurora Kinase	0.73	1.02
RG108	DNA Methyltransferase,Transferase	0.75	1.00
GSK-LSD1 2HCl	Histone Demethylase	0.75	0.95
Valproic acid sodium salt	HDAC	0.77	1.04
Norfloxacin	Topoisomerase	0.77	0.89
Pirarubicin	Topoisomerase	0.77	0.75
Ruxolitinib (INCB018424)	JAK	0.77	0.85
Selisistat (EX 527)	Sirtuin	0.78	0.92
Droxinostat	HDAC	0.78	1.04
AR-42	HDAC	0.79	2.28
Tubastatin A HCl	HDAC	0.79	0.88
GSK2879552 2HCl	Histone Demethylase	0.8	0.85
ENMD-2076	FLT3	1.02	0.87
Tozasertib (VX-680; MK-0457)	Aurora Kinase	2.13	1.02
Gemcitabine HCl	DNA/RNA Synthesis	2.35	1.40
EPZ004777	Histone Methyltransferase	2.36	1.17
CX-6258 HCl	Pim	2.43	0.72
UNC0379	Histone Methyltransferase	2.52	1.51
Tofacitinib (CP-690550) Citrate	JAK	2.59	0.92
Mizoribine	DNA/RNA Synthesis	2.65	1.08
IOX1	Histone Demethylase	2.74	1.00
Procarbazine HCl	DNA/RNA Synthesis	2.87	1.00
SGI-1027	DNA Methyltransferase	2.96	1.24
AZD2461	PARP	3.02	0.87
Panobinostat (LBH589)	HDAC	3.42	1.35
Entinostat (MS-275)	HDAC	3.76	1.15
Dacinostat (LAQ824)	HDAC	3.87	1.92
MC1568	HDAC	5.63	3.04
SP2509	Histone Demethylase	7.08	1.33
SRT1720 HCl	Sirtuin	16.18	0.44
Veliparib (ABT-888)	PARP	45.5	1.17
Filgotinib (GLPG0634)	JAK	57.18	1.06
